# How rural and urban parents describe convenience in the context of school-based influenza vaccination: a qualitative study

**DOI:** 10.1186/s12913-014-0663-5

**Published:** 2015-01-22

**Authors:** Candace Lind, Margaret L Russell, Ramona Collins, Judy MacDonald, Christine J Frank, Amy E Davis

**Affiliations:** Faculty of Nursing, University of Calgary, Calgary, Alberta T2N 1N4 Canada; Department of Community Health Sciences, Faculty of Medicine, University of Calgary, Calgary, Canada; Alberta Health Services & Department of Community Health Sciences, Faculty of Medicine, University of Calgary, Calgary, Canada; University of Calgary, Calgary, Canada

**Keywords:** Parents, Health services accessibility, Immunization programs, Schools, Canada, Alberta, Qualitative Research, Health services research, Community health planning, Rural population

## Abstract

**Background:**

Seasonal influenza vaccine uptake among school-age children has been low, particularly among rural children, even in jurisdictions in Canada where this immunization is publicly funded. Providing this vaccination at school may be convenient for parents and might contribute to increased vaccine uptake, particularly among rural children. We explore the construct of convenience as an advantage of school based influenza vaccination. We also explore for rural urban differences in this construct.

**Methods:**

Participants were parents of school-aged children from Alberta, Canada. We qualitatively analyzed focus group data from rural parents using a thematic template that emerged from prior work with urban parents. Both groups of parents had participated in focus groups to explore their perspectives on the acceptability of adding an annual influenza immunization to the immunization program that is currently delivered in Alberta schools. Data from within the theme of ‘convenience’ from both rural and urban parents were then further explored for sub-themes within convenience.

**Results:**

Data were obtained from nine rural and nine urban focus groups. The template of themes that had arisen from prior analysis of the urban data applied to the rural data. Convenience was a third level theme under Advantages. Five fourth level themes emerged from within convenience. Four of the five sub-themes were common to both rural and urban participants: reduction of parental burden to schedule, reduction in parental lost time, decrease in parental stress and increase in physical access points for influenza immunization. The fifth subtheme, increases temporal access to influenza immunization, emerged uniquely from the rural data.

**Conclusions:**

Both rural and urban parents perceived that convenience would be an advantage of adding an annual influenza immunization to the vaccinations currently given to Alberta children at school. Improving temporal access to such immunization may be a more relevant aspect of convenience to rural than to urban parents.

## Background

In many countries including the United States, Canada, and some members of the European Union, annual seasonal influenza vaccination is recommended or encouraged for healthy children aged ≥6 months [1-3]. In Canada, this vaccine is available with no out-of-pocket cost (vaccine and cost of administering vaccine) for all persons aged ≥6 months through universal public funding, in all but five of the 13 Canadian provinces and territories [4]. The five that do not provide universal public funding provide publicly funded vaccine to specific populations on the basis of age group, occupation, or health status, including children and adults who have chronic health conditions. Despite this, influenza vaccination coverage has been low. In Canada in 2012 among those aged 12–19 years, coverage was 18.2% [5]. In Alberta, which has had universal public funding since 2009, influenza vaccine coverage among healthy children aged 5–8 years was estimated to be 17.1%, and 12.8% among those aged 8–17 years for the 2012–2013 influenza season [6]. Influenza vaccine coverage also varies by rural/urban residence: in Alberta for the 2010–2011 season, vaccine coverage was lower among rural than urban residents, including among children of all ages [7]. One strategy for increasing vaccine coverage among school-aged children is for them to be immunized at school [8]. Parental consent and child assent is required for children to be immunized at school. In Canada, although all provinces and territories deliver other vaccines to children at school [9], influenza vaccine is not usually delivered by this strategy except in a few rural remote areas. Given rural/urban differences in influenza vaccination coverage, increases in vaccine coverage that might be attainable by vaccine delivery at school might be even more important for rural than for urban areas. In prior work [10] we explored urban parents’ perspectives on the acceptability of adding an annual influenza immunization to the immunization program (school based influenza vaccination [SBIV]) that is currently delivered in Alberta schools, and obtained suggestions for structuring such a program. We found that urban Alberta parents perceived one advantage of offering influenza vaccination at school was convenience for families. Convenience is a dimension of health service accessibility [11] that is related to health service utilization. The convenience of delivering vaccine in a school setting may be particularly attractive to rural parents [12]. We wondered if rural parents in Alberta had similar perceptions. We also wondered how rural parents differed from their urban counterparts in how they talked about convenience. In particular, we explored the construct of convenience as an advantage of school based influenza vaccination. We also explored for rural urban differences in this construct.

## Methods

This study reports on the findings from a research project encompassing purposively sampled parents living in rural and urban areas of the Alberta Health Services (AHS)-Calgary zone, with a focus on comparing rural parent data with previously obtained urban data from the same zone. The AHS-Calgary zone includes both urban and rural areas within 150 kilometers (km) of the City of Calgary, Alberta. It includes more than one third of the residents of Alberta, and 49 communities, of which the largest is the City of Calgary (population > 1,000,000). Rural participants were recruited using previously reported methods to recruit urban participants [10]. Forty-eight rural parents participated in nine focus groups (FG). FG are particularly well suited for obtaining data from lay persons on health services issues [13]. We collected data for the rural FG using teleconference (toll-free telephone line), an acceptable method [14] to make the FG accessible to rural participants. The rural FG were held over the period February 9, 2013 to September 3, 2013, using a previously published semi-structured interview guide [10]. Consent forms and the teleconference moderator script were emailed to potential participants a week in advance of the FG and participants were encouraged to contact study personnel before the FG to obtain answers to any questions or concerns they might have. At the beginning of each FG, the moderator asked each participant to confirm their consent to participate, including consent to recording the session. Continued participation in the teleconference FG was understood to be an indication of informed consent. Participants were advised to use only their first names in the conversation to help protect their privacy. The FG were digitally recorded and transcribed verbatim. Participants were sent a post-office money order (first class mail) for $50 after the teleconference; this allowed us to validate self-reported postal codes.

For the purpose of this study, we defined ‘rural’ as residence within the AHS-Calgary zone but more than 50 km from the City of Calgary. We examined indicators of rural residence among participants: postal codes, self-reports of living in town versus in the country, distance from Calgary of community of residence, and population size of the community of residence. In Canada, a postal code with zero in the second position of the six-digit code indicates a rural area that is not accessed by letter carriers [15,16].

### Ethics and role of the funding source

The study was approved by the University of Calgary Conjoint Health Research Ethics Board (Ethics ID# 24083). Participants gave informed consent prior to taking part in the study; the consenting process included information about the researchers and the purpose and rationale of the study. The funders had no role in the design or conduct of the study; data collection, management or analysis or interpretation, or preparation, review, or approval of the manuscript.

### Data analysis

As reported elsewhere [10], data were organized using NVivo10 software. Three team members (CL, MLR and RC) reviewed the rural transcripts using the template of themes developed from the analysis of the nine urban FG. This template organized themes according to the Population Health Promotion Model (PHPM) [17]. The PHPM is depicted as a cube, with three sides representing the social determinants of health, an array of levels for action, and action strategies; all sitting on a base that is its evidence-based decision-making foundation [17]. The model includes population levels (e.g. individual, family, community, system, society) with or for whom actions might be taken, as well as action strategies to improve health. In this case we also explored laterally between rural and urban populations. One strategy to improve health is to reorient health services, for example, by improving health service accessibility. To explore how rural parents discuss ‘convenience’ compared to their urban counterparts, we performed another layer of thematic analysis [18] and coding within the level 3 theme of convenience. First we read the transcripts and each independently came up with a table of subthemes (i.e. a coding scheme) that was discussed until there was consensus on subthemes and exemplar quotes to illustrate them. Then we coded the data. We compared the demographics of participants in the rural FG to those who had participated in the urban FG (cross tabulations, chi-square and Fisher’s exact test for association for categoric variables, Kruskal Wallis statistic for continuous variables; two-tailed alpha = 0.05). Epi Info™ 7.1.2 (CDC 2011) was used for analysis of the demographic data.

As described previously [10], rigour was addressed through confirmability, dependability and triangulation.

## Results

### Participants

A detailed description of participants in the nine urban FG has been published elsewhere [10]. There were 28 participants in the nine rural FG, and the median number of participants per FG was three (range 1–5). All nine rural FG had participants with children in grade levels K-6. Participants in two FG also had children in grade levels 7–9 and in five FG, also had children in grade levels 10–12. Table 1 summarizes the demographics of the rural participants. Participants self-reported living in 16 communities with population sizes ranging from 176 to 26,319. Thirteen of the 28 participants named communities with a population size of 5,000 or less. Although their mean distance from the City of Calgary was 79 km, six of the 16 communities were more than 100 km from Calgary (maximum 150 km). Rural participants were similar to participants in urban FG with respect to distribution of sex, lone parent status, participant vaccinated against influenza, having at least one child who had been vaccinated against influenza or median number of children in family. However, only 17.8% (5/28) of rural participants compared to 39.6% (29/48) of urban participants had a university degree (p = 0.003). Rural FG participants were also significantly younger (19/28, 67.9% vs. 17/47, 35.4% aged 20–39 years, p = 0.009), more likely to have a zero in the second position of their postal codes (12/28, 42.9% vs. 0/48, p < <0.001) and to live ‘in the country’ rather than ‘in town’ (19/28, 32.1% vs. 0/48, p < <0.001) than participants in the urban FG.

**Table 1:** **Description of participants in rural FG (N = 28)**

**Participant attributes**		
	**N**	**%** ^*^
SEX		
Male	3	10.7
Female	25	89.3
AGE GROUP		
20-39 years	19	67.9
40 years or older	9	32.1
LONE PARENT		
Yes	1	3.6
No	27	96.4
LIVES IN TOWN OR COUNTRY		
Town	19	67.9
Country	9	32.1
SECOND DIGIT OF POSTAL CODE = 0		
Yes	12	42.8
No	16	57.2
HIGHEST LEVEL OF EDUCATION ATTAINED		
High school or less	5	17.8
Some post-secondary	5	17.8
Post-secondary or trades certificate	13	46.4
University degree	5	17.8
NUMBER OF CHILDREN IN FAMILY		
1	5	17.8
2	12	42.8
3 or more	11	39.3
Median number of children in family	2.0	___
PARTICIPANT EVER VACCINATED AGAINST INFLUENZA		
Yes	26	92.9
No	2	7.1
AT LEAST ONE CHILD EVER VACCINATED AGAINST INFLUENZA		
Yes	25	89.3
No	2	7.1
Not sure/missing	1	3.6

*totals may not add up to 100% due to rounding.

### Applicability of themes identified from urban focus groups

The themes previously identified among urban parents also applied to data obtained from our rural participants and data saturation was attained. The three broad first level themes included: Advantages (of SBIV), Disadvantages (of SBIV), and Implications for program design and delivery [10]. Under the theme of Advantages there were five second level themes. These second level themes, organized using the PHPM, were populations (child [individual], family, community, sector [system], and society) where advantages could accrue. As observed in the urban FG, convenience was a level 3 theme under the second level theme of Advantages to the family. Finally, under the theme Implications for program design and delivery, there were nine second level themes that exemplified multiple suggestions for structuring a SBIV program from a parent’s perspective.

### Convenience

Many parents in both rural and urban FG used the word ‘easier’ when discussing SBIV: “it would make life easier if it was done in the schools for the children.” (FG 1 urban); “I think it [SBIV] is easier just in the sense that all of the kids are already at school…” (FG 12 rural). School was thus a convenient location for children to be immunized. A consequence of making influenza immunization more convenient might be an increase in vaccine uptake: “I am thinking a lot of parents don’t like the inconvenience of having to make an appointment and go somewhere else. A lot of them probably don’t get their children done. If it were just done in the school, a lot more people would take advantage of that.” (FG 13 rural). “It might be something else that helps people decide whether they have their children vaccinated or not for a yearly flu. Being a single parent, how much flexibility do you have with your work to be able to stay home from work…?” (FG 3 urban).

We found five level 4 subthemes within ‘convenience’. Table 2 displays these with exemplar quotes. Four of these five subthemes emerged in both rural and urban FG. Reduction of parental burden to schedule (Table 2) captured parents’ perception of not having to schedule, track or plan child, family or work activities around scheduling influenza immunization for their children. Reduction in parental lost time relates to parents not having to lose time from work, make extra trips or wait in line-ups for immunization, or lose time to care for children ill with influenza. Parents perceived that delivery of influenza vaccine at school would decrease parental stress by giving them respite from having to manage their children during vaccination. Having children immunized against influenza at school would also increase the number of physical access points for influenza immunization and for some, ameliorate the necessity of having to travel to a different community to access this vaccination. Having a school influenza immunization program would also increase the date and time availability for influenza immunization, thereby increasing temporal access to influenza immunization, a theme that emerged only among rural parents (Table 2).

**Table 2:** **Level 4 themes with exemplar quotes within Level 3 theme ‘Convenience’**

	**Rural**	**Urban**
Reduction of parental burden to schedule	“If it is at school, the children are already there and we can just sign them up and then it is taken care of, rather than juggle schedules between work and children’s activities to get them to those special clinic times.” (FG 10 rural)	“…having four children in three different schools and … in different after school activities, just pinpointing a time where I can take them or have to take them out of school to get their flu vaccination is very hard. Plus I’m a full-time student myself. So it’s kind of like an orchestrated play that we have to do just to get to our vaccination after.” (FG 2 urban)
Reduction in parental lost time	“…it would probably be a great time saver. You don’t have to worry about taking time off work, you don’t have to worry about rushing home from working during the evening, or waiting in long lines with small children…” (FG 10 rural)	“There will be, whether admitted or not, parents who will be happy to have their children have the flu shot because therefore their kids won’t get sick and have to take time off school and therefore they won’t have to take time off work to be with their kid.” (FG 4 urban)
Decrease in parental stress	“…they don’t have to go and take their kid and go through that ordeal of having them cry and being upset. It is done at school, and they don’t have to worry about [it].” (FG 12 rural)	“…then they don’t have to worry about fighting their kids. The screaming, the crying, the no, no, no…” (FG 1 urban)
Increases physical access points for influenza immunization	“…definitely convenience, especially in [community A], because we don’t have a health clinic yet. We do have to make that trip to [community B] or into the city.” (FG 13 rural)	“I think another benefit too if a parent doesn’t drive. If you are new to the country and you don’t drive and you are depending on somebody else to drive you to the flu clinic but yet your children are getting it, you might be able, during the day, to get yourself to a flu clinic; but to take four kids with you on the bus [is a hardship]…” (FG 2 urban)
Increases temporal access to influenza immunization	“…in our community they would only have the flu shot at certain times, like only every Tuesday from 7–9 for three periods in September or October. So if you don’t remember that, then you’ve missed your chance…” (FG 15 rural)	Concept did not arise in urban FG

While both urban and rural data included comments about physical access, rural and urban participants differed in the dimensions of the limitations or challenges they experienced (Table 3). As mentioned, temporal access as a dimension of convenience emerged only among rural parents. Physical access was an issue for both rural and urban parents, however rural parents’ concerns were expressed in terms of a lack of consistent site for influenza clinics (when they existed in their own town) or of having to travel out of town to access influenza immunization. Urban participants discussed physical access in terms of transportation barriers such as not being able to drive or of having to drive in rush hour traffic (Table 3).

**Table 3:** **Unique rural/urban manifestations of convenience subthemes**

	**Concept**	**Exemplar quote**
RURAL	*Temporal access*: Limited hours of operation of influenza immunization clinic	“I know we had one clinic in [Community A] but it was only one night and not everybody could make it out for that time…” (FG 13 rural)
	*Physical Access*: Having to drive to a different town to access an influenza vaccination clinic	“I still have to travel to the next town…which is like 25 minutes away.” (FG 11 rural)
	*Physical Access*: Lack of stability in influenza immunization clinic locations	“…The flu shot was just at a hotel, they set up a walk-in thing. The next year it was at the civic center, or the health unit. It is all over the place.” (FG 17 rural)
URBAN	*Physical Access*: Transportation barriers	“…parents not driving and clinics not being close to them…” (FG 5 urban)
		“… with us we were driving in rush hour … and I hate that.” (FG 6 urban)

## Discussion

This study adds to the current state of knowledge of parent perceptions of potentially using SBIV, by showing that the themes and sub-themes identified from urban parents [10] also apply to data obtained from rural parents: advantages, disadvantages, and implications for program design and delivery; a strength of our study. However, our research also shows that temporal access issues may be of greater relevance to rural than to urban residents.

The demographic characteristics of participants in the rural FG compared to those who participated in the urban FG supports that our purposive sampling to obtain rural participants was successful - they lived outside of the City of Calgary, were from small communities, and were more likely to live in areas not served by letter carriers. Similar to other rural Albertans [19], participants in our rural (compared to urban) FG were less likely to hold a university degree. The purposive sampling that we employed however was not designed to recruit a statistically representative sample of rural and urban Albertans.

The construct of access to care is complex [20]. As noted by Fiedler [21], people require resources (including personal time) to be able to use health care services. Andersen and colleagues [11] defined access as “those dimensions which describe the potential and actual entry of a given population group to the health care delivery system.” They listed subjective indicators for convenience as travel time, travel cost, waiting time, appointment time and cost of visit. Our findings are consistent with this. Participants indicated that having children immunized against influenza at school would reduce the demand on parental resources (e.g. juggling schedules for appointments, travelling to appointments, lost time from work, stress from dealing with distressed children at immunization appointments). They also suggested that addressing these factors might increase uptake of vaccine. Parents also identified physical and temporal access barriers to influenza immunization. It has been noted elsewhere that in Alberta, rural (compared to urban) communities have reduced access to health care in general, as measured by healthcare provider/population ratios [19].

Although we studied parent perceptions of convenience related to vaccination at school, access issues for influenza immunization have been noted for other age groups. Among the elderly, access barriers included distance, convenience of health center locations, hours in which influenza immunization services were available, as well as transportation [22]. Thus perhaps it is not surprising that temporal access was a theme identified from our participants. It is interesting, however, that we observed this only among rural residents. This might be due to sampling (i.e. had we studied a larger urban sample we may have observed this among urban as well as rural participants) or the perception may reflect reality. To further explore this we reviewed unpublished AHS-Calgary zone data for dates, times and places for 2012–2013 public health influenza immunization. We found that urban residents (in two communities) had an average of 366 opportunities (mass clinics, plus clinics by appointment only) to obtain influenza immunization, whereas residents of rural communities (31 communities) had an average of only 22.5 such opportunities.

Barriers to physical access may differ between rural and urban populations [23]. While urban residents who do not have personal vehicles may be able to use mass transit systems, these systems may not exist in rural areas where most people have personal vehicles. This is consistent with the pattern of differences we noted between our rural and urban respondents where urban respondents talked about not driving or the challenges of traffic jams, while rural participants talked about the burden of having to drive themselves to other communities.

Future research that employs designs with statistically representative sampling would be required to determine if rural and urban populations differ with respect to the themes we identified within convenience. This information would be important to influenza immunization health service planners.

## Conclusions

Both rural and urban parents perceived that convenience would be an advantage of adding an annual influenza immunization to the vaccinations currently given to Alberta children at school. Whether urban or rural, parents are bombarded with multiple demands placed upon them and cannot attend to all of them. Convenience facilitates access to immunization in multiple ways. Convenience in this context included reduction in parental scheduling burden, reduction in parental lost time (to schedule immunization or to take children to appointments or care for sick children), reduction in parental stress, and increased physical access to immunization. However, urban parents appeared to conceptualize increased physical access as a reduction in transportation barriers such as not having a car, or dealing with rush hour traffic; rural parents conceptualized it in terms of having to travel outside their communities for vaccination. Temporal access was a theme that arose only among rural parents. Making access to influenza immunization more convenient has the potential to increase uptake of this vaccine.

## Abbreviations

SBIV: School-based influenza vaccination – immunizing children with seasonal influenza vaccine at school
AHS: Alberta Health Services
FG: Focus group(s)
km: kilometers
